# Relation between functional mobility and dynapenia in institutionalized frail elderly

**DOI:** 10.1590/S1679-45082017AO3932

**Published:** 2017

**Authors:** Antonio Vinicius Soares, Elessandra Marcelino, Késsia Cristina Maia, Noé Gomes Borges

**Affiliations:** 1 Associação Educacional Luterana Bom Jesus, Joinville, SC, Brazil.; 2 Faculdade Guilherme Guimbala, Joinville, SC, Brazil.; 3 Universidade do Estado de Santa Catarina, Florianópolis, SC, Brazil.

**Keywords:** Frail elderly, Muscle strength, Geriatric assessment, Health of institutionalized elderly, Idoso fragilizado, Força muscular, Avaliação geriátrica, Saúde do idoso institucionalizado

## Abstract

**Objective:**

To investigate the relation between functional mobility and dynapenia in institutionalized frail elderly.

**Methods:**

A descriptive, correlational study involving 26 institutionalized elderly men and women, mean age 82.3±6 years. The instruments employed were the Mini Mental State Examination, the Geriatric Depression Scale, the International Physical Activity Questionnaire, the Timed Up and Go test, a handgrip dynamometer and a portable dynamometer for large muscle groups (shoulder, elbow and hip flexors, knee extensors and ankle dorsiflexors).

**Results:**

Significant negative correlation between functional mobility levels assessed by the Timed Up and Go test and dynapenia was observed in all muscle groups evaluated, particularly in knee extensors (r -0.65).

**Conclusion:**

A significant negative correlation between muscle strength, particularly knee extensor strength, and functional mobility was found in institutionalized elderly. Data presented indicate that the higher the muscle strength, the shorter the execution time, and this could demonstrate better performance in this functional mobility test.

## RESUMO

**Objetivo:**

Investigar a relação entre mobilidade funcional e dinapenia em idosos frágeis institucionalizados.

**Métodos:**

Estudo descritivo, correlacional conduzido com 26 idosos institucionalizados de ambos os sexos com média de idade de 82,3±6 anos. Os instrumentos utilizados foram o Mini-Exame do Estado Mental, Escala de Depressão Geriátrica, Questionário Internacional de Atividade Física, teste *Timed Up and Go*, dinamômetro de preensão manual e dinamômetro portátil para avaliar grandes grupos musculares (flexores do ombro, flexores do cotovelo, flexores do quadril, extensores do joelho e dorsiflexores do tornozelo).

**Resultados:**

Foi observada correlação negativa significativa do nível de mobilidade funcional avaliado pelo teste *Timed Up and Go* com a dinapenia em relação a todos os grupos musculares avaliados, sobretudo os extensores do joelho (r -0,65).

**Conclusão:**

Foi encontrada significativa correlação negativa da força muscular, sobretudo dos extensores do joelho, com a mobilidade funcional em idosos institucionalizados. Quanto maior a força muscular, menor foi o tempo de execução, o que pode significar um melhor desempenho no teste de mobilidade funcional.

## INTRODUCTION

Population ageing is one of the most significant trends of the 21^st^ century. This phenomenon is progressing fast - particularly in Brazil - and this country is projected to rank sixth in elderly population, in 2020.^(^ ^1^ ^)^ However, the ageing process is associated with physiological and functional changes conducive to compromised mobility and autonomy. Among the disabling changes, the frailty syndrome of the elderly (FS) stands out as a highly prevalent condition in this cohort, with unfavorable outcomes on senior health. Functional decline across multiple organ systems is a major cause of vulnerability and may lead to falls, disability, hospitalization and, in advanced stages, death.^(^ ^2^ ^,^ ^3^ ^)^ The frailty syndrome may be clinically explained by loss of muscle mass (sarcopenia), decreased muscle strength (dynapenia), changes in balance, loss of functional mobility and reduced levels of physical activity.^(^ ^4^ ^)^

Dynapenia reflects the progression of sarcopenia and central nervous system changes and is the first and most important clinical manifestation of FS.^(^ ^5^ ^)^ Sarcopenia and dynapenia are both age-related; however, these entities should be studied separately.^(^ ^4^ ^)^ Dynapenia is a better predictor of disability and death, as compared to loss of muscle mass alone.^(^ ^6^ ^)^

Some studies showed that handgrip dynamometry is the easiest method to measure muscle strength in elderly people, and it has good correlation with both lower limb strength and functional performance.^(^ ^7^ ^,^ ^8^ ^)^ Also, handgrip strength is one of FS clinical diagnostic criteria.^(^ ^2^ ^)^

Lower limb strength is related to accomplishment of routine tasks, such as rising from a chair, going down a flight of stairs and walking at appropriate speed, which correspond to basic abilities associated with functional independence levels.^(^ ^9^ ^)^ Although dynapenia is a systemic process, identification of representative muscle groups may contribute to early recognition of elderly persons prone to FS development.

Functional mobility is strongly related to independence levels in elderly people and may be evaluated using the Timed Up and Go test (TUG), a rapid, practical and user-friendly functional test.^(^ ^10^ ^)^

## OBJECTIVE

To investigate the relation between functional mobility and strength of selected muscle groups in frail elderly.

## METHODS

A descriptive, correlational study developed at *Ancianato Bethesda*, in Joinville, Santa Catarina, between February and November 2015. This study was approved by the Human Research Ethics Committee of *Instituto Superior e Centro Educacional Luterano Bom Jesus*, protocol number 393.274, CAAE: 21681413.6.0000.5365. All participants signed an Informed Consent Term attesting to their volunteer participation in the study.

Potential participants were indicated by *Ancianato Bethesda*’s health team among the 98 elderly residing at the organization. Of these, 30 elderly individuals of both sexes, with mean age of 83.2±6 years and diagnosed with FS, were selected and evaluated. The frailty syndrome of the elderly diagnosis was based on the following pre-established criteria:^(^ ^2^ ^)^ decreased handgrip strength; decreased gait speed; unintentional weight loss; fatigue and decreased levels of physical activity. Elderly persons presenting with three or more, one or two, or none of these diagnostic criteria were classified as frail, pre-frail and non-frail, respectively.

Exclusion criteria were as follows: deficiencies resulting from neurologic diseases, such as dementia and stroke; severe heart diseases or limb amputation; severe visual, auditory and/or vestibular deficiency; and incapacitating orthopedic or rheumatic conditions.

Twenty-six out of 30 participants were included in the study, 18 of whom were women. Even after application of general diagnostic criteria, four participants were excluded due to parkinsonism (one participant), signs of dementia (one participant) or time taken to complete the TUG test below 10 seconds (two participants).

Initial assessment consisted of creation of a medical record including personal data, brief clinical history and a list of 12 associated conditions and/or dysfunctions (systemic arterial hypertension, *diabetes mellitus*, stroke, parkinsonism, heart, lung or kidney disease, obesity, rheumatic disease and visual, auditory and/or vestibular *deficits*), medications on use and associated treatments.

The sample was further screened using the following criteria: TUG test execution time (cutoff value ≥10 seconds);^(^ ^11^ ^)^ absence of significant cognitive *deficit* according to Mini Mental State Examination (MMSE) and/or signs of severe depression based on the Geriatric Depression Scale.^(^ ^12^ ^)^

The TUG test was used for specific assessment of functional mobility,^(^ ^10^ ^)^ since this tool has good intra-rater (ICC-0.95) and inter-rater (ICC-0.98) reliability.^(^ ^13^ ^,^ ^14^ ^)^ The International Physical Activity Questionnaire – short form (IPAC-SF) was used to categorize physical activity levels as low, moderate or high with good reproducibility (r_s_=0.95).^(^ ^15^ ^)^ Muscle mass estimation was based on the equation of Lee et al.,^(^ ^16^ ^)^ for total muscle mass index (TMMI) (5.9 to 9.5kg/M^2^) calculation, according to the following formula:

Total Muscle Mass (TMM) = 0.244.BW + 7.80.H1– 0.098.A + 6.6.S + Et– 3.3

Where TMMI is expressed as TMMI (kg.m^-2^)= TMM/E^2^; BW= body weight (kg); H1= height (meters); A= age (years); S= sex (woman= 0; man= 1); Et= ethnicity (Caucasian= 0, Asian= -1.2; Black= 1.4).

Muscle strength was measured using dynamometry. A handgrip dynamometer (Takei^®^) was used to measure handgrip strength, and a portable multiarticular dynamometer (Chatillon^®^) to measure strength in large upper and lower limb muscle groups. Handgrip strength measurements were made according to the recommendations of the American Society of Hand Therapists,^(^ ^10^ ^)^ while large muscle group measurements (shoulder, elbow and hip flexors, knee extensors and ankle dorsiflexors), as recommended by Andrews et al.^(^ ^13^ ^)^ Both devices were calibrated prior to data collection.

The best out of two measurements of maximal isometric voluntary contraction sustained for 3-5 seconds was recorded per muscle group. The arithmetic mean of muscle group measurements was then calculated. Means were normalized to body weight for isometric strength determination (strength x 9.81/body weight = N/Kg).^(^ ^15^ ^)^

A digital scale with 50g resolution (2096PP, Toledo^®^, Brazil) and a stadiometer measuring 1mm increments (ES2020, American Medical do Brasil Ltda., Sanny^®^, Brazil) were used for body mass and height measurements, respectively.

### Data analysis

Data tabulation and analysis were performed using the GraphPad Prism 6 software^®^; means, standard deviations, minimum and maximum values were determined. Relations between variables analyzed in the study (TUG *versus* strength) were investigated using the Pearson’s correlation test. The level of significance was set at 95% (p<0.05).

## RESULTS

Twenty-six out of 30 participants met the inclusion criteria adopted in this study. Selected participants had low levels of physical activity and did not show signs of dementia or depression according to MMSE and the Geriatric Depression Scale, respectively.

Descriptive statistics (means and standard deviations of individual variables) are shown in table 1. Bilateral handgrip and large muscle group strength measurement were expressed as means. Strength measurements were normalized to body mass to minimize potential effects of anthropometric and/or biotypological features. However, the absolute mean 22.2kgf (±8.6) should also be considered, as it is commonly reported in studies in which this variable is controlled.

**Table 1:** Descriptive statistics for variables analyzed in the study

Variable	Mean	SD
Age	83.2	6.0
Number of conditions*	4.1	1.5
MMSE	26.8	3.7
TUG, seconds	14.8	3.2
Body mass index	26.9	4.4
Total body mass index	7.5	1.9
Handgrip strength, N/kg	3.3	0.9
Shoulder flexion, N/kg	9.4	8.8
Elbow flexion, N/kg	10.5	3.4
Hip flexion, N/kg	9.6	7.9
Knee extension, N/kg	15.6	5.2
Ankle dorsiflexion, N/kg	11.4	7.4

* Twelve conditions were considered, as follows: systemic arterial hypertension, diabetes mellitus, stroke, parkinsonism, heart, lung and renal diseases, obesity, rheumatic disease and visual, auditory and/or vestibular *deficits*. SD: standard deviation; MMSE: Mini Mental State Examination; TUG: Timed Up and Go test.

Correlation coefficients between functional mobility (TUG test) and muscle strength are given in table 2.

**Table 2:** Analysis of correlation between Timed Up and Go test results and strength measurements

Handgrip strength (N/kg)	Shoulder flexion (N/kg)	Elbow flexion (N/kg)	Hip flexion (N/kg)	Knee extension (N/kg)	Ankle dorsiflexion (N/kg)
-0.36	-0.56	-0.58	-0.51	-0.65	-0.56
0.025*	0.000*	0.000*	0.001*	0.000*	0.000*

* p<0.05.

Functional mobility was negatively correlated with strength tests, *i.e.*, the lower the strength, the longer the execution time - and the poorer the performance - in the TUG test. All correlation coefficients were significant; however, knee extensor strength was particularly correlated with functional mobility.

## DISCUSSION

Increased life expectancy is associated with increased age-related vulnerability and disability, which are major causes of institutionalization.^(^ ^1^ ^)^ Health surveillance studies involving 436 elderly individuals and all institutions (nursing homes, long-term care facilities and geriatric clinics) in the municipality of Pelotas, Rio Grande do Sul indicate that elderly persons with advanced age (*i.e.*, aged over 75 years) prevail in these settings.^(^ ^17^ ^)^ Similar findings were documented in the present study, which also involved institutionalized elderly with advanced age - a common epidemiological profile among the elderly.

Four out of 12 associated dysfunctions and/or conditions investigated were more prevalent in this study: visual *deficit*, systemic arterial hypertension, rheumatic conditions and vestibular *deficit*. This clinical profile is actually quite common among elderly people, particularly with advancing age.^(^ ^17^ ^)^ It is worth mentioning some features, such as visual impairment, systemic arterial hypertension (of significance in Brazilian epidemiology),^(^ ^18^ ^)^ rheumatic diseases^(^ ^19^ ^)^ and finally vestibular changes leading to compromised balance and increased risk of falls in elderly persons.^(^ ^20^ ^)^

Elderly individuals included in this study did not show signs of cognitive impairment according to MMSE (*i.e.*, MMSE scores were consistent with participants’ schooling). However, the heterogeneity of Brazilian elementary education and associated regional characteristics must be taken into account, as this may lead to different response profiles in cognitive scales.^(^ ^21^ ^)^

Body changes occur with ageing and can be detected using anthropometric measurements, and body mass index is easily applied. It is an important indicator of nutritional status and a predictor of morbidity and mortality.^(^ ^22^ ^)^ Body mass index is highly correlated with body weight, but poorly correlated with height, and normative values adopted for young adults must be adjusted to elderly patients to account for changes in bone and muscle mass, body fat and water content. Although body mass index is widely used for body composition assessment in clinical practice, the isolated use of this parameter is controversial and not an international consensus. In this study, the values found were considered within normal ranges reported for this age group, as per the recommendations suggested by critical analysis of the literature.^(^ ^2^ ^3^ ^)^

Sarcopenia is a complex, incapacitating process in elderly people. Hence, the TMMI can be used for enhanced investigation of body composition in this cohort, since it includes both anthropometric and demographic data.^(^ ^4^ ^,^ ^24^ ^)^ Total body mass index values documented in this study reflect normal reference values.^(^ ^25^ ^)^

Handgrip strength measurements in this study were similar to values given in previous studies with institutionalized elderly involved in falls.^(^ ^26^ ^)^ As regards strength measurement in large muscle groups, knee extensors were better correlated with functional mobility. The TUG test was used to assess functional mobility in this study; therefore, poor performance in this test can be said to truly reflect greater muscle weakness. Knee extensors are vital for good performance in daily activities.^(^ ^27^ ^)^

Studies investigating handgrip and lower limb strength in 150 community-residing elderly persons revealed strong associations between strength tests and frailty criteria, particularly related to lower limb muscles.^(^ ^28^ ^)^ Similar correlations were observed in women in a study involving 621 non-institutionalized Hispanic North American elderly, in contrast with greater correlation with upper limb muscle strength in men in the same study.^(^ ^29^ ^)^

Some studies suggest that decreased knee extensor strength is a predictor of reduced mobility and functionality with advancing age, leading to greater vulnerability, increased risk of falls and greater dependence on senior care.^(^ ^27^ ^,^ ^30^ ^)^

## CONCLUSION

This study revealed significant negative correlations between muscle strength, particularly knee extensor strength, and functional mobility in institutionalized elderly. Data indicate that the greater the muscle strength, the shorter the execution time in the Timed Up and Go test, or the better the performance in this functional mobility test.

The authors of this study suggest the use of dynamometry for this purpose, and not only handgrip strength, as is often the case in this field, with particular emphasis on strength measurements in large muscle groups using portable dynamometry.
